# Increasing active travel: aims, methods and baseline measures of a quasi-experimental study

**DOI:** 10.1186/1471-2458-14-935

**Published:** 2014-09-08

**Authors:** Ralph Chapman, Philippa Howden-Chapman, Michael Keall, Karen Witten, Wokje Abrahamse, Alistair Woodward, Dylan Muggeridge, Jean Beetham, Mark Grams

**Affiliations:** NZ Centre for Sustainable Cities, and Victoria University of Wellington, Environmental Studies Programme, SGEES, P.O. Box 600, Wellington, New Zealand; Department of Public Health, NZ Centre for Sustainable Cities, University of Otago Wellington, P.O. Box 7343, Wellington South, New Zealand; NZ Centre for Sustainable Cities, and Massey University Whariki, Wellesley St, P.O. Box 6137, Auckland, New Zealand; NZ Centre for Sustainable Cities, and School of Population Health, University of Auckland, 261 Morrin Rd, St Johns, Auckland, 1072 New Zealand

**Keywords:** Methods, Cycling, Walking, Quasi-experimental, Physical activity, Active travel, Attitudes, New Zealand

## Abstract

**Background:**

Policy advisers are seeking robust evidence on the effectiveness of measures, such as promoting walking and cycling, that potentially offer multiple benefits, including enhanced health through physical activity, alongside reductions in energy use, traffic congestion and carbon emissions. This paper outlines the ‘ACTIVE’ study, designed to test whether the Model Communities Programme in two New Zealand cities is increasing walking and cycling. The intervention consists of the introduction of cycle and walkway infrastructure, along with measures to encourage active travel. This paper focuses on the rationale for our chosen study design and methods.

**Method:**

The study design is multi-level and quasi-experimental, with two intervention and two control cities. Baseline measures were taken in 2011 and follow-up measures in 2012 and 2013. Our face-to-face surveys measured walking and cycling, but also awareness, attitudes and habits. We measured explanatory and confounding factors for mode choice, including socio-demographic and well-being variables. Data collected from the same households on either two or three occasions will be analysed using multi-level models that take account of clustering at the household and individual levels. A cost-benefit analysis will also be undertaken, using our estimates of carbon savings from mode shifts. The matching of the intervention and control cities was quite close in terms of socio-demographic variables, including ethnicity, and baseline levels of walking and cycling.

**Discussion:**

This multidisciplinary study provides a strong design for evaluating an intervention to increase walking and cycling in a developed country with relatively low baseline levels of active travel. Its strengths include the use of data from control cities as well as intervention cities, an extended evaluation period with a reasonable response rate from a random community survey and the availability of instrumental variables for sensitivity analyses.

## Introduction

Alongside intensifying global problems of climate change and energy insecurity sits the more local but connected issue of insufficient physical activity, the health effects of which are evident in rising global rates of diabetes and many other non-communicable diseases [1, 2]. Policy advisers are seeking further evidence on the effectiveness of policy measures, such as promoting walking and cycling, that potentially offer multiple benefits, including enhanced health through physical activity, and reductions in energy use, traffic congestion and carbon emissions [3].

Consistent with the dearth of research on the effectiveness of health policy initiatives [4], several systematic reviews have shown that there is little robust evidence concerning effective interventions to increase walking and cycling [5, 6]. The aim of this paper is to describe the design, implementation, strengths and limitations of a quasi-experimental study of an intervention to increase active travel (walking and cycling).

The New Zealand Model Communities Programme (MCP) was jointly funded from mid-2010 by central and local government and provided an opportunity for a ‘natural experiment’. Our study was initially funded by small grants from the two MCP councils and the researchers’ universities, and then in 2012 by central government as part of a competitive research grant. The research group was independent of both the New Zealand Transport Agency, which channelled the MCP’s central government funding, and the two local governments involved in the MCP’s funding and implementation. However, using the principles and practices of partnership [7, 8], we established strong working relationships with the four local governments involved.

## Background

Insufficient physical activity is responsible for an estimated 3.2 million deaths per year globally [2]. Long-term observational studies have reported that individuals who walk and cycle regularly experience lower rates of cardiovascular disease (CVD), cancer, and other diseases [9–11]. However, interventions to promote cycling have not generally resulted in an increase in overall physical activity, or sustained reductions in body mass [12]. Two recent reviews have shown limited evidence for slowing weight gain amongst adults [13], but promising evidence for reducing diabetes [14]. Amongst children and adolescents, walking or cycling to school has been linked with improved cardiorespiratory fitness [15], muscular fitness (equivocal) [16] and lower weight (weak) [17].

New Zealand has high rates of car ownership and an increasing rate of obesity and diabetes. The New Zealand Census and the Household Travel Survey show a decline to around 2006 in active travel and substantial decreases in cycling and walking for children aged 5–14, and in cycling among teenagers, in the last two decades [18, 19]. Recent Travel Survey data suggest that cycling is now starting to increase for larger cities (>100,000 residents), but may still be declining for most smaller cities, and that walking may still be declining.

Research indicates that mode shift towards active travel is difficult to achieve without sustained effort [20, 21]. It is difficult to change long-standing and complex patterns of habitual behaviour in the face of pervasive social, economic and environmental forces such as highway building which maintain the status quo, or support increased car use. In addition, social investment in promoting physical activity, and particularly active travel, is currently limited by a lack of evidence on the nature of the institutional barriers to active travel investment, but it appears that few countries’ institutions effectively integrate public health and urban planning considerations in decision making [11]. Whether this is amenable to integrated national action plan development [22, 23] remains unclear.

Recent reviews have summarised current knowledge about encouraging walking and cycling [21, 24–27]. Ogilvie and colleagues reported that a range of interventions, including publicity campaigns and various engineering measures, have been tested and not been effective in causing a significant shift from car trips to walking and cycling [28]. They later concluded that community-wide promotional activities, in conjunction with improving infrastructure for cycling, may increase cycling by modest amounts, but there is a need for more precise measures of travel activity to assess behaviour change, with a focus on areas without an established cycling culture [12].

A review by Pucher and colleagues concluded that there were clear increases in cycling activity associated with: infrastructure interventions, such as bike-lanes and parking; integration with public transport; education and marketing programmes; bicycle access programmes; and changes in laws related to active transport [21]. They found that targeted interventions have achieved measurable mode shifts in some settings, but concluded that substantial increases in cycling will require a combination of many different interventions including improved physical infrastructure, pro-bicycle educational programmes, supportive land use planning and restrictions on car use. That is, a package may perform better than its constituent parts.

Most of the studies that were included in these systematic reviews were cross-sectional in nature and had significant limitations in their design and measurement. In short, there remain gaps in our understanding of the efficacy and relative importance of interventions to increase walking and cycling. There have been few experimental or quasi-experimental studies [29] and a large US controlled study, with four experimental communities and one control community, detected no significant changes [30]. The iConnect longitudinal study of cycling and walking is being carried out in a number of UK sites, but does not include control sites [31, 32]. In New Zealand, some cross-sectional research exists [33] and in Australia a study is being undertaken to evaluate a cycle system extension in Sydney using a control area and 2-year follow-up, with outcome variables to include quality of life impacts [34]. To our knowledge there are no completed systematic community trials of interventions to increase walking and cycling, which have included the broad range of outcomes we have included in our study.

### Safety concerns and the urban environment

Choice of travel mode is generally influenced by perceptions of safety [35, 36]. As cycling increases, injury rates tend to fall, making cycling safer and providing larger net health benefits [37–39]. It is proposed that cyclists become more visible to motorists as the number of cyclists grows and a higher proportion of motorists are likely to be cyclists themselves and therefore more sensitive to the needs and rights of cyclists [21]. This change tends to lead to public and political support for more investment in cycling and pedestrian infrastructure, but in highly car-dominated cities, small increases in cycling may not achieve the “safety in numbers” effect.

Many aspects of the urban environment influence the attractiveness of walking and cycling: greater numbers of bicycling lanes and pedestrian paths can induce more active travel [21]; and greater street connectivity can improve access and thus induce more active travel [40]. Topography, weather and green spaces influence both active travel and motorised travel [35, 41], but whether there is a link between urban open space and active trips is unclear, with results varying by age group and place [42, 43]. Rates of cycling to work or school fluctuate seasonally, with low rates in winter [44].

### The influence of attitudes and habits

Attitudes consistent with the dominant car culture (e.g. “car drivers pay for the road and should always be given right-of-way”) as well as custom and habit, appear to be associated with the level of walking and cycling [21]. Increasing people’s awareness of the benefits of active travel may help in increasing it, although attitudes and perceptions differ across population groups. For example, a Melbourne study found women were more concerned about risks associated with sharing road space with motor vehicles, and this suggests that better off-road facilities could support cycling uptake among women, who are currently under-represented as cyclists [45].

Another psychological construct that seems to strongly influence walking and cycling is habits, generally defined as goal-directed and automated behaviour [46]. For instance, somebody may automatically always drive to the local store. This habit will only change, when the situation changes substantially, i.e. a new cycle route encourages the person to try cycling, instead of driving, to the local shop. The MCP programme presents a unique opportunity to examine whether habitual behaviours change as a result of infrastructural changes.

Studies on travel mode choice have typically examined habits in relation to car use [47]. The concept of habit does not seem to have been used in research on walking, and in only one study of cycling. That study found that habit strength was the strongest predictor of cycling to work, even after variables derived from the theory of planned behaviour (attitudes towards cycling, social norms in favour of cycling, and perceived ability to cycle) and socio-demographic variables were controlled for [48].

### Social gradients in walking and cycling

Compared with other forms of activity, walking and cycling seem less likely to show a social, cultural or economic gradient, although this varies by place and time. Analysis of national New Zealand Travel Survey data from 2003–2007 does not show a socio-economic gradient in bicycle travel and there is no gradient in walking apart from higher levels of walking amongst people with annual income less than $10,000 [49, 50]. It is desirable that this equality is maintained with new interventions at a city level. One study addressing socio-economic grade in the uptake of cycling, the study of the Six Cycling Demonstration Towns in the UK, found that there was an increase in propensity to cycle associated with the intervention across all socio-economic grades [51]. Both the Marmot Review in the UK [52] and the WHO Commission on the Social Determinants of Health have noted the importance of increasing the proportion of people from low-income communities walking and cycling [53].

## The Model Communities Programme (MCP)

**Figure 1:**
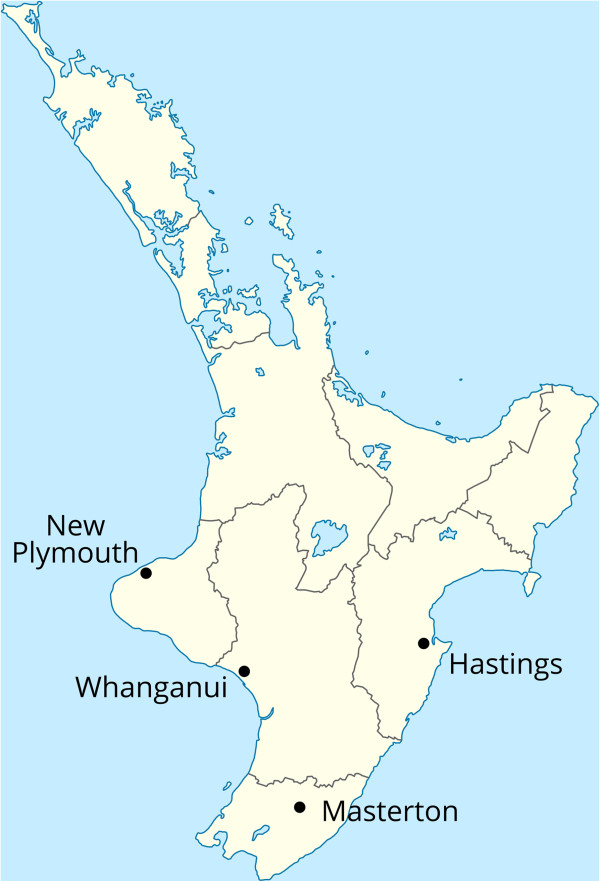
**Location of the four cities in the study, in the North Island of New Zealand (Commons: NordNordWest CC-BY-SA).**

In early 2010 the New Zealand Transport Agency sought tenders from local councils to establish the MCP, with the aim of encouraging the uptake of walking and cycling through structural changes and educational efforts. The MCP aimed to deliver safe, urban environments that would encourage ‘novice users’ to walk or cycle to school or to work in fully integrated walking and cycling transport networks [54]. Two North Island local governments, New Plymouth District Council and Hastings District Council were selected based on the central government’s criteria, and were funded $4.9 million and $4.3 m. respectively from July 2010 to July 2012. New Plymouth and Hastings are small cities of approximately the same population and latitude (Figure 1); both are largely suburban cities, with very few townhouses or apartment buildings. New Plymouth has a lower proportion of its population who are Māori (the indigenous population) (15%) compared to Hastings (24%), but median incomes are similar, as are median ages (Table 1).

**Table 1:** **Socio-demographic and climatic data for the four cities (territorial authorities)**

	Intervention cities		Control cities		
	New Plymouth	Hastings	Whanganui	Masterton	NZ
Estimated resident pop.	71,100	73,200	43,800	23,200	4,184,600
Pop. in 10–19 age-group (%)	15.0	16.2	15.6	15.6	14.9
Median age	38.7	36.6	39.7	40.4	35.9
Māori pop.	10,300	17,800	9,800	4,000	624,300
Māori % of pop.	14.5	24.3	22.4	17.2	14.9
Median income, people 15 & over	22,800	22,600	19,800	21,700	24,400
Mean air temp. August/Feb, °C	10.3/18.0	10.3/19.4*	10.2 /18.5	8.4/17.7	
Mean annual rainfall (mm)	1386	785*	918	928	

*The air temp and rainfall given are for Napier, an immediately adjacent city.

Sources: Statistics New Zealand, 2006 Census data.

The cities developed their programmes in line with local aims and were branded *Let’s Go* in New Plymouth and *iWay* in Hastings, but the strong similarities mean that they can usefully be evaluated side by side. Programme similarities include:

-  Infrastructure upgrading and new investment, e.g. footpath renewal, new tracks, new cycle paths, lighting, bike stands, shared space or pathway projects, etc.
-  Publicity and awareness campaigns for attitudes towards walking and cycling
-  Safety for both communities involves a combination of education and infrastructure investment, e.g. safety education in schools, targeting young people
-  A connectivity and spatial emphasis for key areas such as CBD, schools and residential areas
-  Travel plan support through mapping and internet-based schemes
-  Integration, in the sense of involvement of other government agencies, community groups and schools to help achieve objectives.

The differences between the programmes are relatively minor: New Plymouth includes visitors as a target group; Hastings focuses more on arterial routes (separated shared pathways that link Hastings to surrounding communities); and some sub-programmes are distinct, such as New Plymouth’s ‘dream street’ concept, an initiative to encourage local communities to re-design their street.

## Methods/Design

### The ACTIVE study design

The ‘Activating Communities to Improve Vitality & Equality’ (ACTIVE) study was designed to robustly evaluate the MCP by assessing whether, as a result of the interventions, there is a change in the amount of walking and cycling to work and study (primary outcome), total walking and cycling physical activity, walking and cycling habits, attitudes and awareness. While the intervention cities have undertaken a range of before-and-after traffic and travel counts and surveys, as a way of evaluating their programmes [55], firm causal inferences cannot be drawn from these data in the absence of control data.

As suitably matched control cities, we selected two North Island cities, Whanganui and Masterton (Figure 1), which have similar demographic populations, economic profiles and climates to the intervention cities. The control cities are both interested in promoting active travel, but have not received central government money for this purpose. A study design of this kind sheds light on whether changes observed over time in the intervention cities are likely to be due to the intervention programmes and are not part of a wider trend. This form of quasi-experimental study, while not a randomised control trial, controls for internal validity more than other evaluation methods (such as simple before-and-after studies) and enables some causal inferences to be drawn [56, 57].

**Figure 2:**
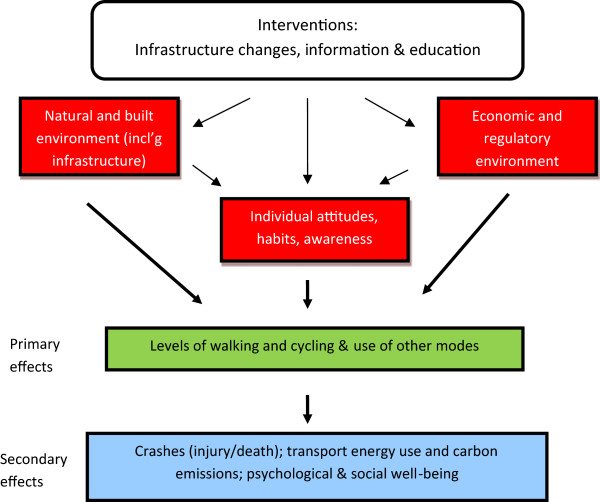
**Schematic diagram of the ‘ACTIVE’ study design.**

We first established a logic model to ensure that we had correctly identified the mediating and outcome variables [58] (Figure 2). Evaluations of behaviour change programmes are strengthened when not only the outcomes, but also possible mediating factors that lead to the behaviour change, are measured and correlate with the main outcome measure.

The primary aim of our study is to assess whether the MCP interventions result in an increase in walking and cycling to work or study, and an increase in physical activity (including recreational walking and cycling). Secondary aims are to:

-  assess whether changes in the number and proportion of active travel trips within the intervention communities varies between areas with different levels of socio-economic deprivation, taking into account access distance to MCP infrastructure;
-  assess whether there is a mode shift from car use, and any change in public transport use, alongside any increase in active travel;
-  assess changes in pedestrian and cyclist injury and death rates associated with the intervention;
-  measure any change in mode-related habitual behaviour, awareness of informational and media campaigns, and attitudes towards cycling/walking, among those who did and did not increase their use of walking and cycling during the study period;
-  assess the MCP’s overall costs and benefits (including estimated health benefits, and energy and carbon savings from mode substitution).

### Settings

While the control cities have substantially smaller populations than the intervention cities, according to the 2006 Census, the socio-demographic characteristics of the populations in the four cities are similar (Table 1). As the four cities are relatively close together geographically, the climatic data are also similar, although annual rainfall in the intervention cities averaged 18% higher than in the control cities.

The transport characteristics of the four cities at the 2006 Census (prior to the intervention) were also relatively similar (Table 2); for example, the percentage cycling to work ranges from 2.0% (New Plymouth) to 3.3% (Whanganui). As they are all provincial cities with ample car parking, the proportion of Census respondents using a bus for the journey to work is very low, at 0.3 - 0.4%; and access to two or more vehicles per household is common (44% in Whanganui to 56% in Hastings).

**Table 2:** **Travel to work, and access to motor vehicles, for the four cities**

	New Plymouth	Hastings	Intervention cities (weighted average)	Whanganui	Masterton	Control cities (weighted average)	New Zealand
Walked or jogged (%)	5.3	4.0	4.6	5.3	5.2	5.3	5.3
Cycled (%)	2.0	2.7	2.4	3.3	2.8	3.1	1.9
Public bus (%)	0.4	0.3	0.3	0.3	0.3	0.3	3.0
Households with access to 2 or more motor vehicles (%)	51.8	55.7	53.8	44.0	49.2	45.8	54.1

Sources: Statistics New Zealand. 2006 Census data.

### Sampling frame

Power calculations were as follows. Assuming a 60% response rate and then 60% retention on follow-up, it was estimated that 1,200 households would need to be sampled, with equal numbers in the intervention and control cities. In the control cities, the proportion of non-cyclists at baseline who are active cyclists at follow-up could be expected to be about 2%. Based on expert opinion and the Demonstration Towns study in the UK [51], we anticipated that the intervention could increase this proportion in the combined intervention cities to 6%, which could be detected from an attained total sample size of 430 (215 households per group) with 80% power and Type I error probability associated with the null hypothesis of 0.05. To allow for a reduced effective sample size due to clustering of respondents at the household level, the sample size was conservatively calculated as though there were only one response per household, even though all household members 10 years of age and over were asked to participate in the Household Survey.

The random sample drawn was based on lists of addresses of ratepayers supplied to us by the four district councils (territorial authorities), limiting the selection to the urbanised parts of the four districts. We focused on these areas since the intervention infrastructure investment is focused in the urban parts of the districts. The Census data for the four districts relate to a greater rural area than the urban areas from which we drew our sample, so we would expect some differences between Census averages and our sample baseline variable averages. Intervention households were randomly selected from strata defined by areas close to, and distant from, MCP infrastructure, and by low and high socio-economic deprivation mesh-blocks.

**Figure 3:**
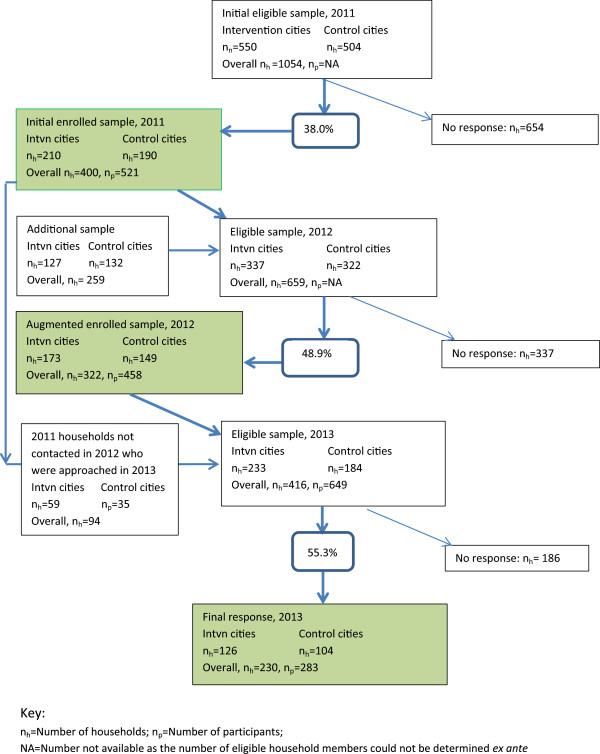
**ACTIVE study sample flow diagram.**

The response rate for the baseline Household Survey carried out over winter 2011 was 38.0% (the number of households with completed questionnaires divided by the number of eligible households, after sample loss due to houses not being occupied, or found to be commercial premises, etc.). Sample numbers and response rates are shown in Figure 3, and described further below, but in short, 521 participants in 400 households were surveyed in 2011. In 2012, combined with a supplementary sample of 127 households, there was a 48.9% response rate, giving 322 households (458 people). In 2013, there was a response rate of 55.3% of households, giving a final year sample of 230 households (283 respondents).

### Data collection method

Sample households were initially sent an introductory letter and information sheet which asked them to participate in a study which involved them being interviewed about their walking, cycling and transport habits and views, and general well-being. Children aged 10 years and over were given a specially tailored and simplified information sheet. We sought consent, including from children in the presence of their parents, and in all cases, agreement to complete the face-to-face interview was taken as consent. All participants were interviewed at home and children were interviewed in the presence of their parents. Parents were able to refuse if they did not want their children to participate. Ethical approval for the study method was granted by the University of Otago Human Ethics Committee (Category A –involving children– 11/107).

Baseline survey data were collected first in each city in mid-2011. Interviews were carried out by teams of trained students from the local polytechnics and experienced interviewers. Follow-up interviews were undertaken in the next two years, 2012 and 2013, at the same time of year. In the 2013 survey we employed all professional interviewers.

To offset sample loss after the baseline year, some additional households were added to the randomly drawn sample in 2012 and followed up in 2013, adding sample for the longitudinal comparisons (2011–2013, 2011–2012, 2012–2013). Additional approval was obtained from the Ethics Committee to go into a draw for a NZ $300 grocery voucher. As specified *a priori* in our study design, the data from the two intervention cities will be combined in the analysis to gain sufficient statistical power. Changes in travel behaviour in these cites over the same interval will be compared with the combined data from the control cities.

The distribution of the respondents in the four cities in the ACTIVE baseline survey sample (Table 3) can be compared to the distribution in the 2006 Census (Tables 1 and 2). There is some sampling bias toward female respondents and older respondents, compared with the Census. This may be due to women being more available to respond to surveys, and younger people (below 20 years) being significantly less often at home when interviewers called, although up to three call-backs were made by interviewers. However, low income groups were proportionally represented; for example, 39% in the intervention and 43% in the control cities respectively had personal incomes below $20,000, while at the Census, 50% had incomes below $22,000 (weighted median in Table 1 is $22,000). No important divergence from the Census was found for ethnicity (a sample average of 18.7% were Māori, while an average of 19.8% were Māori at the Census).

**Table 3:** **Baseline information for the intervention and control cities**

	New Plymouth	Hastings	***Intervention cities total***	Whanganui	Masterton	***Control cities total***
Individual socio-demographic factors at baseline (2011 sample + 2012 additions)						
No. of persons responding (2011 plus new in 2012)	209	195	*404*	222	137	*359*
Age (% < 20)	11%(22/202)	19%(36/190)	*15%* *(58/392)*	16%(31/200)	11%(14/125)	*14%* *(45/325)*
(% 60+)	33%66/202)	22%(42/190)	*28%* *(108/392)*	33%(66/200)	33%(41/125)	*33%* *(107/325)*
Sex (% female)	60%(125/208)	63%(122/195)	*61%* *(247/403)*	61%(136/222)	64%(86/135)	*62%* *(222/357)*
Ethnicity (% Maori)	15%(24/165)	21%(39/182)	*18*% *(63/347)*	20%(33/168)	19%(20/104)	*19%* *(53/272)*
(% European)	75%(123/165)	69%(126/182)	*72%* *(249/347)*	76%(127/168)	79%(82/104)	*77%* *(209/272)*
Personal income (% up to $10,000/y)	18%(33/186)	18%(29/158)	*18%* *(62/344)*	16%(31/191)	19%(20/103)	*17%* *(51/294)*
(% $10,001-$20,000/y)	22%(40/186)	21%(33/158)	*21%* *(73/344)*	27%(51/191)	24%(25/103)	*26%* *(76/294)*
(% > $40,000/y)	32%(60/186)	35%(55/158)	*33%* *(115/344)*	28%(54/191)	30%(31/103)	*29%* *(85/294)*
**Transport behaviours at baseline (2011 sample only)**						
% trips to work (last 7 days) - walked or ran	11%(35/310))	4%(11/274))	*8%* *(46/584)*	10%(32/316)	13%(29/232))	*11%* *(61/548)*
- cycled	0%(0/310)	8%(21/274)	*4%* *(21/584)*	4%(12/316)	8%(18/232)	*5%* *(30/548)*
- bus	0%(0/310)	2%(5/274)	*1%* *(5/584)*	0%(0/316)	0%(0/232)	*0%* *(0/548)*
- car or other	90%(278/310)	86% (237/274)	*88%* *(515/584)*	86%(271/316)	80%(185/232)	*83%* *(456/548)*
Access to modes% access to a bicycle	53%(69/131)	59%(76/128)	*56%* *(145/259)*	43%(65/152)	60%(62/104)	*50%* *(127/256)*
% access to a car	91%(119/131)	94%(120/128)	*92%* *(239/259)*	79%(120/152)	92%(96/104)	*84%* *(216/256)*
% with physical disability	15%(19/131)	14%(18/128)	*14%* *(37/259)*	16%(24/152)	11%(11/104)	*14%* *(35/256)*
**Physical activity behaviours at baseline (2011 sample only)**						
Hrs walking (mod + vigorous) last 7 days	3.6	4.4	*4.0*	3.2	3.5	*3.4*
Hrs cycling (reg + vigorous) last 7 days	0.25	0.80	*0.53*	0.20	0.64	*0.42*

Transport behaviours at the 2011 baseline are also reported in brief (trip to work only, for comparability with the 2006 Census) in Table 3. The sample’s proportion walking or running to work was 8 and 11% (intervention and control cities), compared with 5% for both pairs of cities at the Census, which may reflect the greater rural area in the Census. Similarly, the proportion of trips to work by bicycle was 4% and 5% (against 2% and 3% at the Census) and by bus was 1% and 0% (0% and 0% at the Census).

To assess whether mode choices were influenced by lack of access to modes such as bicycles, we investigated access to bikes and cars, and presence of a physical disability among respondents. The 2011 baseline data showed that 56% and 50% (intervention and control cities) reported having access to a functioning bike, while 92% and 84% had access to a car. Overall some 14% reported a physical condition that prevented respondents walking or cycling in the last 7 days. Questions to describe baseline walking and cycling activity were based on IPAQ conventions [59]; these showed that at the baseline, such activity had a higher prevalence in the intervention than in the control cities (18% more walking in the intervention cities; 26% more cycling).

Supplementary data included: an intercept survey of cyclists in Hastings and New Plymouth, and qualitative data from focus groups and interviews in Hastings. The purpose of the intercept survey was, following Ogilvie [28], to enable a more nuanced evaluation of whether any increased active travel arises from new people participating, or people who were already walking/cycling at the beginning of the MCP increasing their activity. A clearer picture of this is potentially significant for assessing the value of the benefits of the MCP. A qualitative study in Hastings explored the factors influencing people’s active travel, especially the factors influencing their walking and cycling behaviour patterns, and their awareness of *iWay* at an early stage in the programme.

### Outcome measures

Although the main outcome measure was derived from survey data on longitudinal changes in individual travel and mode choice, secondary data sources were also available. The key outcome variables were numbers and distances walking or cycling to work or study, and time spent in walking and cycling physical activity. Multi-level data were collected at city (district council), household and individual level (Table 4). As we were able to code the geographical position of each respondent’s house, we were not only able to quantify the distance of each individual to their nearest upgraded cycle-way/walkway, but also to their reported workplace/school.

**Table 4:** **Outcome and other variables and their levels**

Level	Source	Variable	Measures
***Outcome measures***			
City	Ministry of Health	Road crash injuries	Hospitalisations (no.)
			Deaths (no.)
	Selected schools	Mode of travel to school	Hands up in school’ surveys: Frequency by mode (no.)
Individual	Face-to-face survey	Distance and time by journey purpose	Work/study/shopping/leisure/accompanying family or friends (km, minutes)
		Distance and time by mode	Walk-run/cycle/bus/car/other (km, minutes)
		Physical activity (walking)	Days moderate/vigorous walking in last 7 (no.)
			Time taken, moderate/vigorous walking (hrs)
		Physical activity (cycling)	Days regular/vigorous cycling in last 7 (no.)
			Time taken, regular/vigorous cycling (hrs)
			Frequency of cycling, last 4 weeks/12 months (no.)
		Public transport use	Frequency, last 4 wks/12 months (no./yes or no)
		Attitudes affecting mode choice	Rating
		Habits, perceptions of modes	Mode choice for regular activities, ratings
		Awareness of MCP	Yes/no; Source of awareness; participation
		Well-being in recent weeks	SF36 short form ratings
***Independent variables and potential confounders***			
City	NZ Census 2006, 2013	Population growth	People (no.)
		Average personal income	Personal income before tax ($)
	District council	Traffic levels	On-road traffic counts (cyclists, pedestrians, cars) (no.)
	NZ Census 2006	Small-area socio-economic deprivation	NZDep index
Household	Face-to-face survey	Modal access	Access to a functioning bicycle (yes/no)
			Access to a car (yes/no)
	Google Maps	Distance to infrastructure	Home-infrastructure distance (km)
	http://www.walkscore.com	Accessibility to local destinations	Walkability score (index)
	http://www.walkscore.com	Accessibility to CBD	Transit score (index)
Individual	Face-to-face survey	Distance to work	Home-work distance (km)
		Distance to study	Home-study distance (km)
		Age	Years
		Ethnicity	Group
		Sex	M/F
		Personal income	Personal income before tax ($ band)
		Employment status	Category (Student/Worker/Seeking work/Looking after home or family/Retired/Other beneficiary)
		Physical condition	Disabling physical condition in last 7 days (yes/no)

The individual household survey data were supplemented by a before-and-after ‘hands-up’ school survey of mode use. With assistance from the councils involved, we accessed road survey data of vehicle, cyclist and pedestrian counts conducted before, during and after the intervention.

## Data analysis and methodological issues

The main level of analysis will be at the level of the city (intervention vs control), mediated by individual exposure to the infrastructure component of the MCP interventions. This exposure is partially determined by geographic factors such as where individuals live, work, study; and how and where they travel, as well as their exposure to traffic. This, in turn, reflects individual demographic and lifestyle factors such as age, sex, social class, income, job, and mode of travel, influencing the way people lead their daily lives.

The follow-up measures will enable calculation of any changes in active travel behaviour attributable to the intervention, as well as any increase in relative inequalities in active travel behaviour. We will fit multi-level regression models on the available survey data on travel behaviour, which include national data being collected at the same time as the survey described here. The models will estimate individual change in mode choice using the data at the household level, extrapolated to the intervention and control cities. As the construction of the infrastructure continued over the period spanned by the survey, a factor in the model will measure whether any changes in the intervention cities relative to the control cities continued incrementally with time.

Secondary analyses will be carried out on outcomes including attitudes to travel, counts of cyclists and pedestrians, and road crashes (injury/death).

## Discussion

Our quasi-experimental ACTIVE study is attempting to increase the robustness of evaluation of walking and cycling programmes, by using a stratified random sample of participants in matched intervention and control cities, most of whom have been followed up for two years. The sample appears reasonably aligned with the 2006 Census: although low-income and younger people are under-represented.

Our baseline measures show that, as expected, driving was the dominant mode; indeed driving (or being driven) was the preferred option for all types of trips, except going to the local park. This preference for car travel does not seem to be predominantly influenced by access to bikes as, though almost everybody had access to a car, over half the sample had access to a bike. A minority of people (14%) reported a physical condition that prevented them from walking or cycling in the last seven days.

There are a number of strengths to this quasi-experimental study. The study has sufficient power to potentially detect a significant change in cycling and walking behaviour overall, and enable cautious causal inferences to be drawn about the efficacy of the programme. We collected the survey data at approximately the same time each year, during winter/early spring. As these seasons are not ideal for cycling and walking, this may exert a downward effect on active travel levels.

The random sample was classified to let us take account of potential confounders such as socio-economic status. Instrumental variables (the distance of a respondent’s dwelling from the major aspect of the intervention, the new structural cycleways and walkways) were measured to enable their potential use in sensitivity analyses.

New Zealand cities’ cycling and walking cultures have weakened over the last few decades, although as noted above, in some larger cities there has recently been an increase in cycling. The focus of this study on four smaller cities, all with relatively low levels of walking and cycling, allows us to illuminate the potential for change starting from a low base and facilitates the generalisation of our results to a range of societies dominated by car travel.

Nonetheless, there are a number of important methodological issue in the study, the first being our choice of control cities. As Ogilvie et al. [5], p.122 noted, ‘It may… be unrealistic to aspire to anything more than “broadly comparable” control areas’ in such studies. While our intervention city samples differ in certain respects from the control city samples, they match them closely in other significant respects, including socio-demographically and ethnically. There were some differences in travel behaviour, with slightly more people walking to work in the intervention cities than in the control cities. On the whole, however, the matching provides confidence that when we later compare the results from the control and intervention cities, the differences are not due to major inter-city differences. A further strength of our study is that we can use other survey data collected by the New Zealand Travel Survey over the study period to supplement the control data collected.

A difficult issue in relation to transport evaluation studies is when to begin collecting baseline data. [5]. Although MCP infrastructural works in principle started from mid-2010, in practice they took time to get underway, so that little had changed on the ground by the time baseline data were collected in mid-2011. However, some promotional activities had taken place, and this may have contributed to some ‘baseline awareness’ of the two MCP programmes by mid-2011, and possibly a diminishing of the extent of measured changes in behaviours. However, in each year of our study, we measured the participants’ awareness of the programmes; for example in mid-2011, only a minority of respondents were aware of the *iWay* programme.

Other authors of studies in the transport, health and environment area have noted difficulties with low recruitment or response rates (e.g. Saelens et al., [24], p.1557, referring to 15-20% rates); this, and on-line recruitment of samples, may limit generalizability of findings. However, response rates were relatively high in our study compared to existing studies and we collected longitudinal data, which will be more robust to non-response bias than cross-sectional data.

Our research relationship with local authorities was important. Most district council officers were very helpful, but in a control city, council cycling survey data were not provided, despite reminders. Consequently, a consistent set of cycling data from on-road surveys from all areas studied is not available. Our relationship to the councils also related to the practical, funding issues inherent in evaluating a natural experiment. We were not aware of the MCP until the money had been allocated, which meant that our study was initially very constrained for funds. There were two major consequences on the selection of outcome measures and the choice of interviewers. We focused on proxy health outcomes, such as levels of physical activity, rather than independent health indicators, such as BMI or cardiovascular functioning, largely because of the financial resources available. Similarly, although our use of local polytechnic students, who largely matched the socio-demographics of the cities they lived in, may have strengthened research capacity in these areas and enabled us to begin in a timely fashion, it may have reduced our overall response rate; this was boosted in the final year when we were able to employ professional interviewers. This change in interviewers’ experience over the three rounds of the study may also have introduced some instrumentation bias. However, such bias will be the same for both treatment and control cities, so it should not affect the comparisons in travel behaviour that we will estimate.

## Conclusion

Data collection in the ACTIVE study has been successfully concluded. Multivariate analyses will include an assessment of the role of socio-demographic variables in explaining changes in levels of active travel to work or study, and physical activity. The data collected will allow comparisons over time and between intervention and control cities, to establish the extent to which the MCP is having an impact on active travel behaviour, and walking and cycling physical activity. We will also be able to establish whether the programme has been successful in significantly affecting crash outcomes, changing travel habits and attitudes, improving reported well-being, and raising public awareness of walking and cycling support.
